# Thermoluminescence as a Research Tool to Investigate Luminescence Mechanisms

**DOI:** 10.3390/ma10121357

**Published:** 2017-11-26

**Authors:** Adrie J. J. Bos

**Affiliations:** Luminescence Materials, Faculty of Applied Sciences, Delft University of Technology, 2629JB Delft, The Netherlands; a.j.j.bos@tudelft.nl; Tel.: +31-(0)15-278-4705

**Keywords:** thermoluminescence, trap depth, energy level, rare earth ions, persistent luminescence, band gap engineering, tunnelling, photosynthesis, thermal quenching

## Abstract

Thermally stimulated luminescence (TSL) is known as a technique used in radiation dosimetry and dating. However, since the luminescence is very sensitive to the defects in a solid, it can also be used in material research. In this review, it is shown how TSL can be used as a research tool to investigate luminescent characteristics and underlying luminescent mechanisms. First, some basic characteristics and a theoretical background of the phenomenon are given. Next, methods and difficulties in extracting trapping parameters are addressed. Then, the instrumentation needed to measure the luminescence, both as a function of temperature and wavelength, is described. Finally, a series of very diverse examples is given to illustrate how TSL has been used in the determination of energy levels of defects, in the research of persistent luminescence phosphors, and in phenomena like band gap engineering, tunnelling, photosynthesis, and thermal quenching. It is concluded that in the field of luminescence spectroscopy, thermally stimulated luminescence has proven to be an experimental technique with unique properties to study defects in solids.

## 1. Introduction

In recent decades, the search for new luminescent materials has been intensified due to applications in many different fields, like light emitting diode (LED) based lighting systems, persistent luminescence phosphors, storage phosphors, scintillators, and up and down conversion materials for solar cells. Progress in the identification of new luminescent materials requires insight into the underlying mechanisms and the role of the defects in those materials. Achieving this insight requires instrumentation and dedicated experimental methods.

Thermally stimulated luminescence (TSL) is light produced by heating a solid to a temperature below that of incandescence. The light is only observed after exposure of the solid to radiation, i.e., absorption of energy from an external source. The heat only works as a trigger. The main application of TSL is in radiation dosimetry since it has been shown that for some materials, the emitted light is proportional to the absorbed dose to which the material has been exposed. This is the case not only in personal dosimetry, but also for environmental, clinical, and high dose dosimetry. Another important application is using TSL as a dating method complementary to radiocarbon. TSL is able to date inorganic materials, mainly ceramics, while radiocarbon is limited to organic materials. TSL dating has also turned out to be useful in fields different from archaeology and historical architecture, in particular, the dating of sediments.

However, it was recognized in the fifties of the last century that TSL can not only be used for dosimetry and dating, but also for other purposes like measuring the efficiency of surface catalysts and determining impurities in rocks [1]. Thermoluminescence is extremely sensitive to defects in the material under investigation and can therefore also be used to study these defects. As the instrumentation developed further, it became clear that TSL could also be used in material science [2,3]. Recently, Mihóková and Nikl [4] reviewed spectroscopic methods, including TSL, to probe the excited state of emission centers. However, a review especially dedicated to the role of thermoluminescence as a research tool for the investigation of luminescent materials is lacking. This review briefly recalls the theoretical background and instrumentation and then uses a range of examples to show how thermoluminescence studies can be effective in revealing the luminescent mechanism for a variety of research problems.

## 2. Theoretical Background

In order to understand how thermoluminescence (TL) can be used to study luminescent materials, a theoretical background of the phenomenon is indispensable. The following discussion borrows heavily from references [5,6,7,8,9].

The basic effect leading to the production of TL is the trapping of charge carriers, i.e., electrons and holes, produced during exposure to an external source at defect sites in the material. Defect sites can be divided into two categories: (1) those inherently present in the material; and (2) those produced by external means, such as deliberately doping the sample with impurities. A well-known example of the first category is a negative ion vacancy. Such a vacancy can trap an electron and is then called an *F* center. An example of the second category is a lattice vacancy caused by a higher valence impurity ion at the position of a lattice ion. A trivalent cation impurity in a divalent lattice, for example, would induce the formation of a cation vacancy in the lattice to maintain charge neutrality. Cationic vacancies are potential sites for trapping holes. There are many other types of defects that can act as electron or hole trapping centers. Revealing the nature of those defects is part of the research of luminescent materials.

Heating of the material causes the release of the trapped charge carriers and the recombination of electrons and holes at a luminescent center. The release of some of the stored energy excites the center and relaxation may lead to the emission of light. The nature of the luminescent center can be revealed by measuring the emission spectrum which is in many cases characteristic for a specific element. We note that not every recombination event leads to luminescence. If, for example, there is not enough energy available to excite the center, the recombination will be non-radiative. The process leading to recombination includes, in many cases, the transition of charge carriers through the conduction or valence band, but localized transitions may also take place. The theory of TSL consists of solving the relevant sets of coupled differential equations, describing the charge transfer. In the following, we discuss the simplest model. This model shows all the characteristics of the TSL phenomenon.

### 2.1. Simple Model and Characteristics of TSL

**Figure 1:**
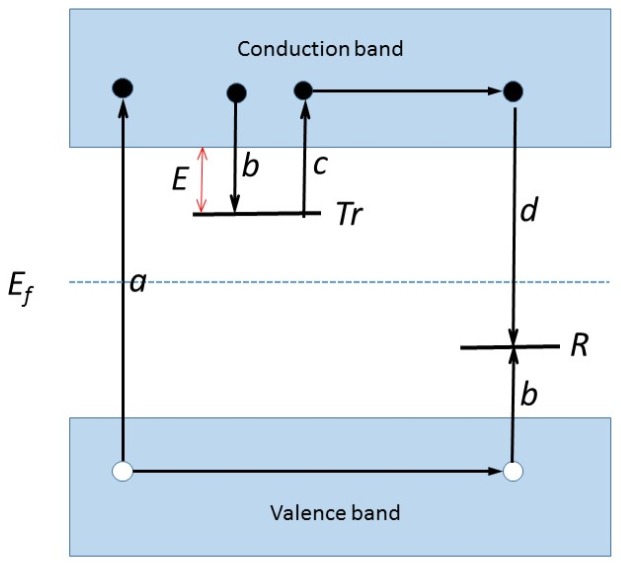
A simplified model of the thermoluminescence mechanism: During the excitation stage (**a**) electrons and holes are produced which are trapped (**b**) in trapping, *Tr*, and Recombination centers, *R.* During the heating stage (**c**) electrons are released to the conduction band and move through the Conduction band until they encounter a hole in a recombination center (**d**). The recombination energy will excite the center and relaxation of the excited center will produce the luminescence. For explanation of *E* and *E_f_* see text.

The simplest model to explain TL is the One Trapping centre One Recombination (OTOR) model (Figure 1). Absorption of radiant energy with energy greater than the band gap results in the ionisation of valence electrons, producing energetic electrons and holes which will, after thermalization, produce free electrons in the conduction band and free holes in the valence band. A certain percentage of the freed charge carriers will be trapped: the electrons at *Tr* and the holes at *R* (transitions *b*). There is a certain probability that the charge carriers escape from these traps due to thermal stimulation. The probability per unit time of release of an electron from the trap, *p*, is assumed to be described by the Arrhenius Equation (1):

(1) $$p=s\exp\left(-\frac{E}{kT}\right)$$

The pre-exponential factor *s* is called the frequency factor or attempt-to-escape factor. In the simple model, the factor *s* is considered as a constant (not temperature dependent) with a value in the order of the lattice vibration frequency, namely 10^12^–10^14^ s^−1^.

*E* is called the trap depth or activation energy, which is the energy needed to release an electron from the trap into the conduction band (see Figure 1). The other symbols have their usual meaning; *k* = Boltzmann’s constant = 8.617 × 10^−5^ eV/K, and *T* is the absolute temperature. The quantities *E* and *s* are called the trap parameters. Their values determine whether the electron will escape at a certain temperature *T*. If the trap depth *E* >> *kT*_0_, with *T*_0_ representing the temperature at irradiation, then any electron that becomes trapped will remain so for a long period of time, so that even after exposure to the radiation, there will exist a substantial population of trapped electrons. Furthermore, because the free electrons and holes are created and annihilated in pairs, there must be an equal population of trapped holes at level *R*. Because the normal equilibrium Fermi level *E_f_* is situated below level *Tr* and above level *R*, these populations of trapped electrons and holes represent a non-equilibrium state. 

The return to equilibrium can be speeded up by raising the temperature of the TL material above *T*_0_. This will increase the probability of detrapping and the trapped electrons will now be released from the trap into the conduction band. The charge carrier migrates through the conduction band of the material until it undergoes recombination at recombination center *R*. In the simple model following Randall and Wilkins [10,11,12], this recombination center is a luminescent center where the recombination of the electron and hole leaves the center in one of its higher excited states. Return to the ground state is coupled with the emission of light quanta, i.e., thermoluminescence. However, it should be realized that not every de-excitation leads to the emission of light. The non-radiative transitions depend on the temperature. Sometimes, at higher temperatures, the TL is completely thermally quenched. In the simple model, it is assumed that there is no quenching and no retrapping, i.e., all electrons released into the conduction band give rise to recombination under the emission of light. Let us denote *n* (m^−3^) as the concentration of trapped electrons and *m* (m^−3^) as the concentration of holes trapped at *R*. Then, the TL intensity *I*(*t*), in photons per unit volume and per unit time (m^−3^ s^−1^) at any time *t* during heating is proportional to the rate of recombination of holes and electrons at *R*. This rate of recombination is, under the mentioned assumptions, equal to the rate of thermal excitation of electrons from *Tr* into the conduction band:

(2) $$I(t)=-\frac{dm}{dt}=-\frac{dn}{dt}=np=ns\exp\left(-\frac{E}{kT}\right)$$

This differential equation [2] describes the charge transport in the lattice as a first-order process. Usually, TL is observed as the temperature *T* is raised as a linear function of time according to:

(3) $$T(t)=T_{0}+\beta t$$

with *β* (K s^−1^) representing a constant heating rate and *T*_0_ indicating the temperature at time *t* = 0. Solving Equation (2) with this temperature profile leads to the well-known Randall-Wilkins equation:

(4) $$I(T)=-\frac{1}{\beta }\frac{dn}{dt}=n_{0}\frac{s}{\beta }\exp\left(-\frac{E}{kT}\right)\exp\left[-\frac{s}{\beta }\int_{T_{0}}^{T}\exp\left(-\frac{E}{kT^{\prime }}\right)dT^{\prime }\right]$$

with *n*_0_ representing the total number of trapped electrons at *t* = 0 and *T′* indicating a dummy variable. This equation describes the shape of a peak. Such a peak shape can be understood as follows: as the temperature increases, the intensity initially increases because of the detrapping of the trapped charge carriers and the consequent recombination which initiates luminescence, after which point the intensity reaches a maximum and finally decreases as the number of charge carriers becomes depleted. The shape and position of the peak maximum are governed by the trap parameters (material constants) and the heating rate (readout parameter).

**Figure 2:**
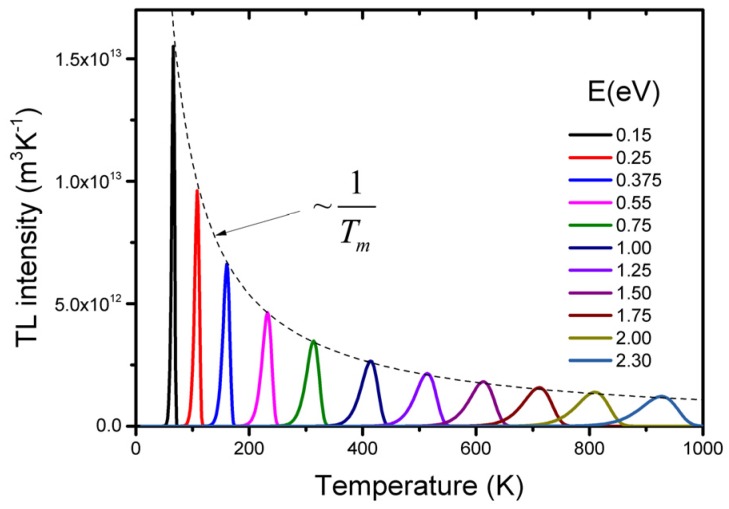
TL glow peaks calculated with the Randall-Wilkins Equation (4) for *n_0_* = 10^14^ m^−3^; *s* = 10^11^ s^−1^, *β* = 1 K/s and different values of the trap depth *E*.

**Figure 3:**
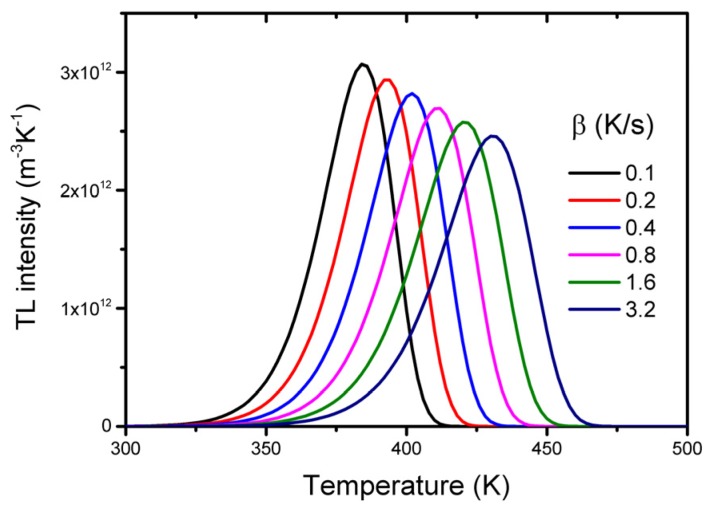
TL glow peaks calculated with the Randall-Wilkins Equation (4) for *n*_0_ = 10^14^ m^−3^; *s* = 10^11^ s^−1^, *E* = 1 eV and different values of the heating rate β.

In Figure 2, the shape of the so-called glow peak according to Equation (4) is shown for different values of the trap depth *E*, while the other parameters are kept constant. We see that for deeper traps: (*i*) the glow peak temperature shifts to a higher temperature which corresponds with the thought that the deeper the trap, the more energy needed to release the charge carrier; and (*ii*) the luminescence is spread out over a wider temperature interval resulting in a broader glow peak. The total intensity, the area of the glow peak equal to *n*_o_, remains the same since this value only depends on the absorbed dose delivered before the readout. The shift of the glow peak temperature shows the sensitivity of the TL method: a difference in trap depth of only 0.05 eV gives a difference in glow peak temperature of an easy to measure 20 K (for the parameter values mentioned in Figure 2). The intensity *I_m_* at the temperature of the glow peak maximum, *T_m_*, can be described by [13]:

(5) $$I_{m}(T_{m})=n_{0}\frac{\beta E}{kT_{m}^{2}}\exp(-g_{m})\sim \frac{n_{0}}{T_{m}}$$

with *g_m_ = g(E/kT_m_)* and *g = g(E/kT)*, an exponential integral with a value very close to unity for *T* = *T_m_* is seen. As we will see below, the ratio *E/T_m_* is almost constant so that with Equation (5), we deduce that *I_m_*~*n_0_/T_m_*. For deeper traps (increase of *T_m_*), the height of the glow peak decreases while the area under the curve (~*n_0_*) remains the same, resulting in an increase of the width of the peak. The shape of the glow peak also depends on the heating rate with which the TL material is read out (see Figure 3). The temperature of the maximum in terms of trap parameters *E* and *s* and the heating rate *β* can be found by equating the derivative of Equation (4) to zero which yields:

(6) $$\frac{\beta E}{kT_{m}^{2}}=s\;\exp\left(-\frac{E}{kT_{m}}\right)$$

From this equation, it follows that the glow peaks are shifted towards higher temperatures when the heating rate is increased. This can be understood as follows. A TL glow peak results from two factors, which change in opposite directions when the time elapses, i.e., the temperature increases, resulting in: (*i*) an increased escape probability and so a higher rate of recombination, leading to an increased number of photons emitted; and (*ii*) a decreased number of charge carriers available for recombination. At a lower heating rate, more time elapses during the same temperature increase, allowing more recombination events to occur, so that the charge pairs (electrons and holes) are eliminated faster and the TL maximum is reached at a lower temperature. From Equation (6), it can be derived that the relation between *T_m_* and the heating rate *β* can be approximated by:

(7) $$T_{m}=a+b\ln\beta$$

with *a* and *b* representing constants depending on the activation energy *E* and frequency factor *s*. From this relation, it follows that the shift of *T_m_* with the heating rate is only pronounced if the heating rate is changed significantly. A typical value is 10 K for any doubling of the heating rate. For deeper traps, it is more than for shallow traps. For lower frequency factors, it is also higher. 

Calculation of the trap depth from given *T_m_* and *s* is hampered by the fact that Equation (6) is transcendental and the equation should be solved by iteration. However, Equation (6) can also be re-written as:

(8) $$E=\frac{s}{\beta }\left\{T_{m}\;\exp(-\frac{E}{kT_{m}})\right\}kT_{m}$$

It appears that in a wide range of trap depths and for a fixed ratio *s/β*, the product {*T_m_* exp(−*E/kT_m_*} is constant within a few percent. Thus, for a given *s* and *β*, the trap depth *E* is quite accurately proportional to *T_m_*. This is illustrated in Figure 3, where *E* is plotted against *T_m_* for a wide range of values of the ratio *s/β*. We see that in good approximation we can write:

(9) $$E=C_{U}\;kT_{m}$$

**Figure 4:**
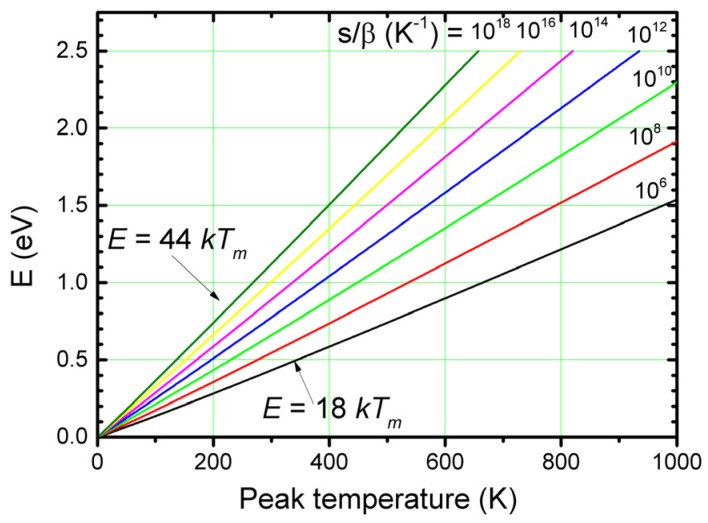
Trap depth *E* calculated with Equation (6) as a function of the peak temperature for a wide range of values of the ratio *s*/*β*.

The constant of proportionality *C_U_* is called the Urbach constant, since Urbach [14] assumed that, no matter the sample, we can use *E*(*eV*) = *T_m_*(*K*)/500 = 23 *kT_m_*. Although dimensionless, *C_U_* is not a constant. From Figure 4, we see that *C_U_* varies between 18 and 44 depending on the ratio *s/β*. The numerical value of the Urbach constant shows that an average thermal energy of 3/2 *kT* with *kT* as the lattice vibrational energy, is by far not enough for an electron to escape from the trap. However, the energy is distributed according to a Maxwell-Boltzmann distribution. The high value of the Urbach constant means that only a small number in the tail of the Maxwell-Boltzmann distribution exceeds the energy needed to escape. It results in a sensitivity of the peak maximum position for variation of the trap depth between 26 K and 64 K per 0.1 eV. 

Thermally stimulated luminescence is also sensitive in the sense that the number of trapping centers needed to detect TL is low. Defect densities as low as 10^7^ cm^−3^ can produce a measurable TL signal, whereas techniques such as Electron Paramagetic Spectroscopy (EPR) and Optical Absorption Spectroscopy (OAS) are only sensitive for higher defect concentrations of 10^12^ cm^−3^ or more [2,15]. Finally, we like to mention that thermoluminescence is a phenomenon that can be observed from materials in various appearances. It is not restricted to single crystalline material. Non-crystalline solids can be used as well. Because of the high sensitivity, only 20 mg of material is usually enough to produce a measurable signal. These properties make TSL a very useful technique in material research.

### 2.2. Deviations from the Simple Model

The simple model is not realistic. In real TL materials, the number of trapping states is, in general, more than one. Some of these states are related to intrinsic defects of the host material, whilst other centers are dopants deliberately added during the synthesis. Within one material, there could also be different luminescent centers present, for example, *F*-centers and dopants in the form of lanthanides ions. As a consequence, the charge traffic in the material will be much more complicated than depicted in Figure 1. We will only briefly describe some possibilities. For a deeper and quantitative treatment, the reader is referred to textbooks [6,7,8,9].

In the simple model, it is assumed that in the heating stage, the electron is released to the conduction band where it moves relatively freely until it encounters a hole center where it recombines. A possibility that is studied extensively in the literature is that the released electron has two options: recombination followed by luminescence or to be retrapped by a vacant trap center. It can be shown that retrapping leads to non-first-order kinetics and this will affect the shape of the glow curve. A typical property of non-first-order behavior is that the glow peak maximum shifts to a lower temperature as the absorbed dose, i.e., the number of produced free electrons and holes, increases. Not only the maximum shifts, but the entire shape of the glow curve, changes with an increasing dose. However, the experience of many years of study of TL materials showed that for many materials, the temperature of the glow peak maximum of individual glow peaks remains practically constant over a wide dose range, and there are no systematic changes in the glow curve shapes with the irradiation dose. Sunta [9], page 71, states that, “In fact as far as our experience goes, we have not come across any material which shows a non-first-order behaviour by peak shift change in dose.” Lewandowski and McKeever have given a physical justification for why a first-order process dominates in nature using a generalized description of thermally stimulated processes. Pagonis and Kitis [16] have shown that the presence of many competitive processes during the heating stage of TL may be correlated to the remarkable stability of the glow curve shapes exhibited by most materials, and to the prevalence of first-order kinetics. 

The prevalence in nature of first-order kinetics does not mean that every glow curve can be described with Equation (4). The simple model of Figure 1 assumed that the trap depths associated with localized states are single-valued in energy. In real materials, the trap depths associated with particular defects can be spread over a range of values, i.e., they tend to be distributed, rather than discrete. The result is that the glow peak is the sum of a large number of first-order glow peaks which emerges as one broad glow peak. The idea of a distribution of trap depths is as old as the theory of TL itself, as first suggested by Randall and Wilkins [10,11,12]. They showed that such distributions exit in calcites and dolomites. Quasi-continuous distributions of trap depths may be expected in non-crystalline materials, as a result of the large number of possible surroundings of the defects. Examples have been given for feldspars [17], Ca–Be rich aluminosilicate (Bavenite) [18], and chariote silicate, a gemstone material [19]. They have also been presented for synthetic materials for dosimetry like CaSO_4_:Dy [20], Li_2_B_4_O_7_:Cu [21], Al_2_O_3_:C [22] and persistent luminescent phosphors like SrAl_2_O_4_:Eu,Dy [23], SrAl_2_O_4_:Eu [24], CaAl_2_O_4_:Eu,Nd [25], Zn*_x_*Ga_2_O_3+*x*_:Cr^3+^ [26,27], Ca_6_BaP_4_O_17_:Eu^2+^,R^3+^ (R = Dy, Tb, Ce, Gd, Nd) [28], and Ba_2_Si_5_N_8_:Eu^2+^ [29], as well as for scintillators GAGG:Ce and LuAG:Pr [30].

Another deviation from the simple model that should be mentioned involves the number of centers. In general, a real TL material will show more than one single electron trap. Not all these traps will be active in the temperature range in which the sample is heated. It could be that electrons in the deeper trap are unaffected. However, their trap filling will affect the TL signal. Something similar can be said for the number and behaviour of recombination centers. 

In principle, it is possible to set up a model with different traps and recombination centers and write down the rate equations that describe the charge traffic. But it is clear that the more traps and recombination centers are involved, the more parameters, like trapping and recombination probabilities and the concentration of trapping and recombination states, will be necessary. Determining all these parameters from the shape of the TL glow curve is tedious work.

### 2.3. Methods for Extracting Trap Parameters

A number methods have been developed for evaluating the activation energy from the shape of the TL peak. The simplest one is in the case where the frequency factor *s* is already known from another experiment like Raman spectroscopy. By determining *T_m_* from the glow curve and using Equation (8), the trap depth *E* can then easily be calculated. When studying the effect of various dopants in a host material, this method is an easy and powerful method. Examples are given by Lecointre et al. [31] for YPO_4_:Pr^3+^,Ln^3+^ (Ln = Nd, Er, Ho, Dy) and Luo et al. [32] for RE_2_O_2_S (RE = La, Gd, Y, and Lu).

Another method involves the shift of the maximum temperature *T_m_* while changing the heating rate *β*. Hoogenstraaten [33] suggested measuring the glow curve for several heating rates. Considering Equation (6), it can be seen that a plot of ln(*T*^2^_m_/*β*) versus (1/*T_m_*) should yield a straight line, from the slope of which, *E/k*, the activation energy *E* can be determined. Although this method has been shown to be valid for the first-order situation, it has been demonstrated that this method is rather general. Since the shift of *T_m_* varies with ln*β* (see Equation (7)), one should vary the heating rate over several orders of magnitude to get an accurate result. In applying this method, one should also realize that during TL readout with readers using contact heating, the temperature of the sample differs from the measured temperature of the heating element. Kitis and Tuyn [34] have developed a method to approximate this so-called temperature lag based on:

(10) $$T_{mj}=T_{mi}-c\ln\left(\frac{\beta_{i}}{\beta_{j}}\right)$$

where *T_mj_* and *T_mi_* are the maximum temperatures of the glow peak at heating rates *β_j_* and *β_i_*, respectively, and *c* is a constant. The constant *c* can be approximated by *c* = (*T_m_*_2_ − *T_m_*_1_)/ln(2), where *T_m_*_2_ and *T_m_*_1_ are the temperatures corresponding to heating rates 1 and 2 K/s, i.e., two heating rates where the lag can be neglected. The temperature lag Δ*T* = *T_mj_* − *T_mi_* can be used to correct the temperature at higher heating rates. The lag can be considerable. For a 0.9 mm thick LiF sample measured at a heating rate of *β* = 6 K/s, the temperature lag is already 14 K [35].

A method suggested by Garlick and Gibson [36] is called the “initial-rise” method. If we consider Equation (2), at the low temperature end of the peak, *n* varies only slightly, and so we can write:

(11) $$I(T)\sim \exp\left(-\frac{E}{kT}\right)$$

A plot of ln(*I*) as a function of 1/*T* in the low temperature ranges should yield a straight line, the slope of which is −*E/k* from which *E* can be calculated. The method is independent of the kinetic order of the process. The main problem here is that the initial-rise range is limited. Pagonis et al. [37] take *I* to be smaller than about 15% of the maximum TL intensity. Chen en Haber [38] use the 5–10% intensity range. In this range, the intensity is low and this may be a significant restriction. But the method is simple and can be successfully used to extract trap depth distributions [25].

A method that has been used extensively is the curve-fitting method. Generally speaking, one compares the shape of the measured TL peak with a theoretical curve, like Equation (4), with the appropriate number of parameters. Horowitz and Yossian [39] have provided an extensive and detailed review of the subject. One has to guess the number of overlapping peaks in a given glow curve in order to perform the analysis. Either first-order or general-order kinetics must be assumed, and the deconvolution program minimizes the difference between the experimental and generated curves, thus yielding the trap parameters of the individual peaks. However, one should be aware that often, the computer produces fit parameters which have no relation with the physical quantities one likes to extract. In the first place, one has to realize that the trap parameters *E* and *s* are highly correlated. This implies that several pairs of *E* and *s* produce almost the same fit. Van Dijk [40] calls the glow curve deconvolution problem, from a numerical point of view, ill-conditioned. For a single first-order peak, the least squares function is, in the *E* − ln(*s*) plane, a very narrow valley of which the bottom is only slightly curved. This was recently experimentally confirmed by Stadtmann and Wilding [41] for the glow peaks of LiF:Mg,Ti. Secondly, if several overlapping glow peaks are involved, the glow curve analysis method assumes that the principle of the superposition is valid. This is only the case for first-order glow peaks. Non-first-order processes imply interaction between the trapping centers which are not taking into consideration by fitting with the sum of a number of non-first-order glow peaks. Thirdly, in a real material, there are usually more trapping centers involved. Chen et al. [42] have shown by simulation that for a model with three trapping centers and one kind of recombination center, two overlapping TL glow peaks may occur, which together look like a first-order peak, but with an anomalous low evaluated trap depth and frequency factor. In a similar simulation, extremely high trap depths and frequency factors could be found for a model with one trapping state and three recombination centers, one radiative and two non-radiative [43]. These simulations show that that one has to be very cautious in the interpretation of the fit parameters. In many cases, they characterize the glow curve under certain experimental conditions but do not reflect kinetic properties of the TL traps.

To determine all the trapping parameters is practically not achievable. However, it should be noted that for many cases, the energy level, i.e., the trap depth, is the most important one. This parameter plays a special role among the other parameters. Due to its appearance in the Arrhenius exponent, the shape and the position on the TL glow peak depends vary strongly on this parameter. One can determine the value of *E* with a good accuracy without knowing the other parameters. For an example, see page 506 of [7].

## 3. Instrumentation

The three main components of TL recording equipment comprise a sample chamber with a temperature controlled heating system, a light detector, and signal processing equipment. The level of sophistication of the components depends on the application. 

The heart of the reader is the heating system. It should provide reproducible and controllable heating profiles. The usual mode of heating is by passing a high current through a thin, low impedance strip of tantalum or platinum, the planchet. The sample (powder of solid chip) is placed in direct contact with the planchet, the temperature of which is monitored via a thermocouple spot welded at the bottom of the planchet. To ensure close thermal contact between the sample and the planchet, the sample is heated in an inert gas atmosphere while the heating rate is below 1 K/s. Most applications of TL, like in dosimetry, are applied above room temperature (RT). This will prevent the observation of TL of very shallow traps which are normally empty at RT. Low temperature measurements may reveal defect centers which play an important role, for example, in scintillators [30]. For low temperature TL measurements, the sample chamber is a cryostat with a heating system that operates in a vacuum. To ensure good thermal contact is, in this case, even more difficult. Irradiations must be carried out at a low temperature and in a vacuum, and thus a window (typically a thin Be window) must be mounted on the cryostat. TL is also used for the study of photosynthesis in intact leaves, and algal and cyanobacterial cells [44,45]. These studies require a very specific instrumental feature: the possibility to cool the sample well below 0 °C, while a vacuum is not possible [46]. 

**Figure 5:**
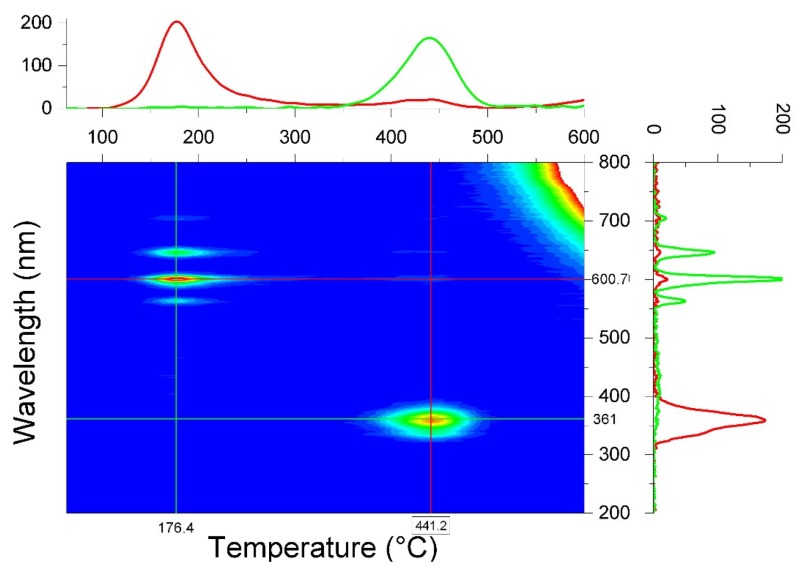
Contour plot of TL intensity as a function of temperature and wavelength of YPO_4_:Ce^3+^,Sm^3+^ after 9.6 kGy ^60^Co irradiation and read out at 5 °C/s. The figure on the top shows two glow curves in red and green for, respectively, emission wavelengths of 601 nm (Sm^3+^ emission) and 361 nm (Ce^3+^ emission). The figure on the right shows two emission spectra in red and green at, respectively, 441 °C and 176 °C. The TL intensity at high temperatures and long wavelengths is from black body radiation. [52].

The second important component of the TL reader is the detection of the light emitted during the heating. In material research, it is essential to measure the emission spectrally resolved, i.e., with a spectrometer [47,48,49,50,51]. The result is a so-called 3D TL plot: the TL intensity as a function of temperature and wavelength. Figure 5 shows an example. The contour plot reveals that the low temperature glow peak mainly originates from Sm^3+^ emission, while the high temperature peak stems from Ce^3+^ emission. If the emission center is known, one can measure the light with a much more sensitive photomultiplier tube (PMT) through a filter that transmits the wavelength or wavelengths of the emission center.

Excitation of the sample can be performed with various irradiation sources: radioactive sources, an X-ray generator, and UV-VIS lamps. Sometimes, the source is integrated in the TL reader which makes the automation of a series of TL readouts possible. Important information can be extracted by measuring the so-called TL excitation spectrum. In this case, the sample is excited with monochromatic light obtained from a UV-VIS source coupled to a monochromator. After excitation with a certain wavelength, the sample is heated and the TL recorded. This measurement is repeated for a series of different excitation wavelengths. By plotting a selected glow peak area as a function of the excitation wavelength (corrected for variations in lamp spectrum), the TL excitation spectrum for that glow peak is obtained [53].

TL-readers equipped with all sorts of possibilities in excitation and detection are commercially available. A well-known reader is the Risø-reader. Recent developments of that reader are described by Thomson et al. [54] and Lapp et al. [55,56]. Another luminescence measurement system—lexsyg—which can be used in fundamental material research, is developed by Freiberg Instruments [57,58,59].

## 4. Thermoluminescence as a Research Tool

The characteristics of a luminescent material strongly depend on the position of the energy levels of the defect levels with respect to the bottom of the conduction band and the top of the valence band. The position of the defect levels also controls whether those defects can act as an electron donor or electron acceptor, or as a hole trap or electron trap. Accurate information on the location of defect levels within the band gap is not easy to obtain. In the following, we will give a series of examples where TSL measurements can help to locate these levels, first from the defect levels of the widely used lanthanides and next from transition metals.

### 4.1. Energy Level Positions

**Figure 6:**
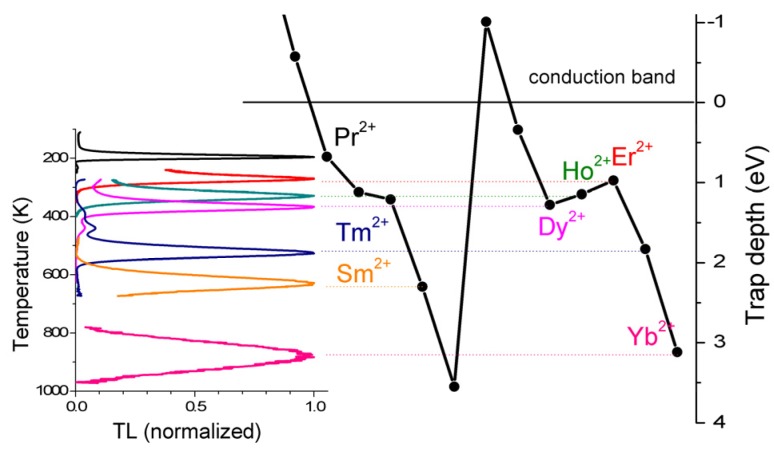
Comparison of predictions of the trap depth of the energy level scheme (the zigzag curve) and experimentally measured glow peak maxima. The codopant and corresponding glow peak have the same color.

Lanthanides are widely used as luminescence activators in inorganic compounds. Dorenbos [60,61,62,63] has developed a phenomenological model predicting the absolute location of *4f* and *5d* states of lanthanides in different compounds. The model predicts all *4f* and *5d* ground state levels of both the divalent and trivalent lanthanides in a compound with only three parameters. With those level positions, one can predict the behaviour of the centers. For example: when the divalent lanthanide *4f^n^* ground state levels are below the conduction band, the corresponding trivalent ions may act as electron-trapping centers. As a function of the type of lanthanide codopant, there is predictable variation in the trap depth as given by a so-called zigzag curve. This behaviour was proposed by Dorenbos in 2005 [64] and later it was experimentally confirmed by TL studies of YPO_4_:Ce^3+^,Ln^3+^ (Ln = Pr, Nd, Sm, Dy, Ho, Er, Tm, and Yb) by Bos et al. [65] (see Figure 6).

**Figure 7:**
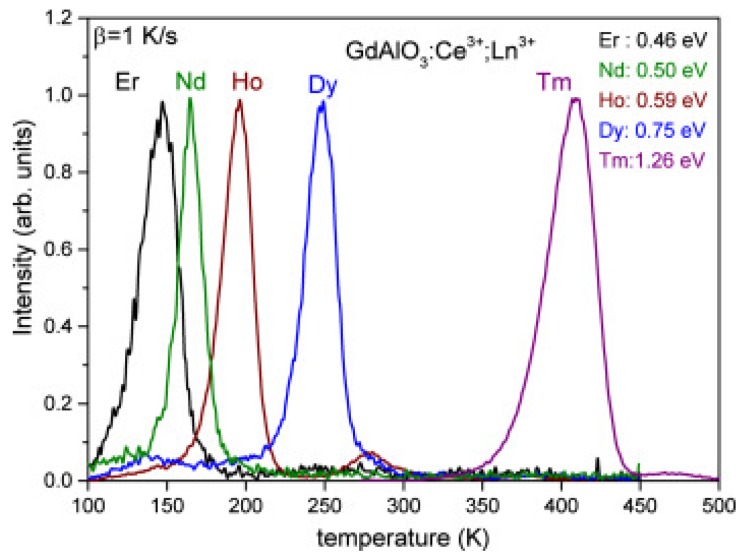
Normalized thermally Stimulated luminescence glow curves of GdAlO_3_:1% Ce^3+^,1% Ln^3+^ (Ln = Er, Nd, Ho, Dy, and Tm). All glow curves were recorded by measuring the Ce^3+^ emission. Data obtained from Luo et al. [71].

Here, Ce^3+^ acts as a hole trapping center and recombination (luminescent) center, while the various lanthanide co-dopants act as electron-trapping centers that trap electrons during the exposure to ionizing radiation. Ln^3+^ ions substitute the Y^3+^ ion. Because Ln^3+^ ions and Y^3+^ are both trivalent cations, no charge compensation is required. The trapping site for all Ln ions is therefore similar and so is the escape frequency *s*. For the same heating rate, the glow peak maximum temperature can be considered as directly proportional to the trap depth (conform Equation (9). If we replace Ce^3+^ in this host material by Pr^3+^ with lanthanides as codopants, the same results are obtained with Pr^3+^ as the luminescent center [31]. A similar study on the energy level positions was performed on GdAlO_3_:Ce^3+^,Ln (Ln = Pr, Er, Nd, Ho, Dy, Tm) (see Figure 7) and lanthanides in Y_3_Al_5_O_12_ [66], LuPO_4_ [67], and Sr_3_AlSi_1−*x*_ O_5_ [68]. In all those cases, the position of the maximum of the glow peak could be connected to the trap depth. This energy level position could be connected to the Charge Transfer band in optical excitation spectra, confirming the systematic trends of the lanthanide energy levels found in the literature [60]. Today, it is well-established that the same shape of the zigzag curve always reappears in inorganic compounds. This property is used to design new dosimetric materials not on a trial and error base, but by systematic investigation [69,70].

**Figure 8:**
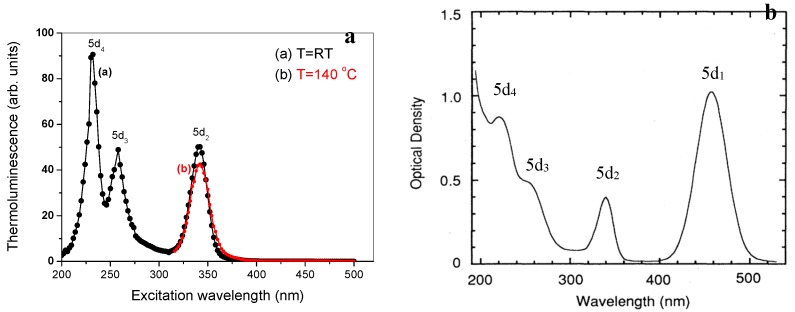
Thermoluminescent excitation spectrum (**a** from You et al. [72]) and optical absorption spectrum (**b**, adopted from Hamilton et al. [73]) of YAG:Ce^3+^. Note the absence in the *5d*_1_ band in the TLES spectrum.

TSL measurements can also help in determining the location of the *5d* levels of lanthanides relative to the bottom of the conduction band and the top of the valence band. This will be clarified with an example for the energy levels of Ce^3+^ and Yb^3+^ codoped Y_3_A_l5_O_12_ (YAG) [72]. In this well-known material, Ce^3+^ is the luminescent center. The co-dopant Yb^3+^ act as an electron trapping center. Thermoluminescence excitation spectroscopy (TLES) was used to establish the location of the energy levels of Ce^3+^. (see Figure 8a). 

There are three strong bands peaked at 342 nm (3.63 eV), 258 nm (4.81 eV), and 232 nm (5.34 eV) in the TLES with the illumination performed at room temperature (RT) (curve (a)). According to the optical absorption spectrum of YAG:Ce^3+^ [73] (Figure 8b), the three bands correspond to absorption bands of Ce^3+^. The fact that a TLES signal is observed implies that there is a significant *5d*_2_ electron escape probability to the conduction band. From this, we conclude that the *5d*_2_ level must be inside the CB or very close (<0.1 eV) below the bottom of the CB. Meanwhile, the *5d*_1_ state of Ce^3+^ is ascertained to lie below the bottom of the CB because no peak at about 457 nm (2.715 eV) is observed in the TLES.

The TLES with the illumination performed at an elevated temperature of 140 °C is also presented in Figure 8a (curve (b)). This temperature was chosen to be lower than *T_m_* in order to avoid thermal cleaning of the TL signal. After illumination, the sample was cooled down to RT and the TL glow curves were recorded. There is still no peak related to the ^2^*F*_5/2_→*5d*_1_ transition for the higher temperature measurement, confirming that the *5d*_1_ state of Ce^3+^ lies well below the CB.

**Figure 9:**
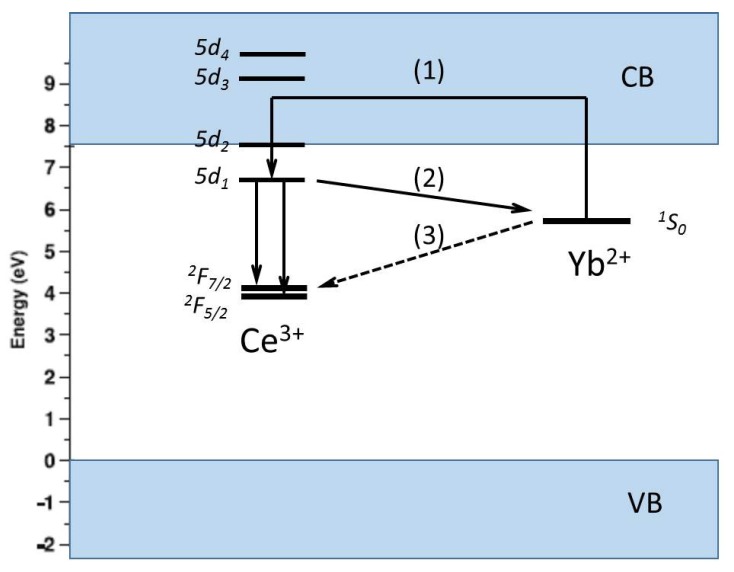
Energy level location of Ce^3+^ and Yb^2+^ in YAG. Pathways (1), (2), and (3) illustrate three different energetically possible electron transfer pathways to and from Ce and Yb in YAG (Adopted from You et al. [72]). CB = Conduction Band and VB = Valence Band.

The TL glow curve showed one main glow peak. With the variable heating rate method, a trap depth of 1.7 eV was determined. The energy level diagram of YAG:Ce^3+^,Yb^3+^ constructed from the derived energy locations is shown in Figure 9. This scheme provides important information on the energetically possible electron transfer pathways to and from Ce and Yb in YAG. The position of the ^1^S_0_ ground state of Yb^2+^ is about 1.7 eV below the bottom of the CB, indicating that Yb^3+^ is a stable electron trap in YAG. Upon β-irradiation, Yb^3+^ captures an electron from the CB, becoming Yb^2+^, while Ce^3+^ changes into Ce^4+^ by trapping a hole from the VB. During TL, the trapped electron is thermally excited from Yb^2+^ to the CB. It is captured by Ce^4+^, resulting in an excited *5d*_1_ state of the Ce^3+^, as illustrated in pathway (1). Finally, the characteristic *5d*→*4f* emission of Ce^3+^ is observed as a TL glow. The position of the *5d*_1_ level with respect to the conduction band gives an explanation of the thermal quenching of photoluminescence. The position of the same *5d*_1_ level with respect to the ^1^*S*_0_ level of Yb^3+^ makes pathway (2) possible. This non-localized transition competes with pathway (1), explaining why the TL increases with an increasing heating rate. Pathway (3) finally explains the fading behaviour of the TL. 

Other examples in which TSL measurements are used to determine the position of the *5d* excited levels with respect to the conduction band of Ce^3+^ are given by Babin et al. [74] and Wu et al. [75].

### 4.2. Persistent Luminescence

**Figure 10:**
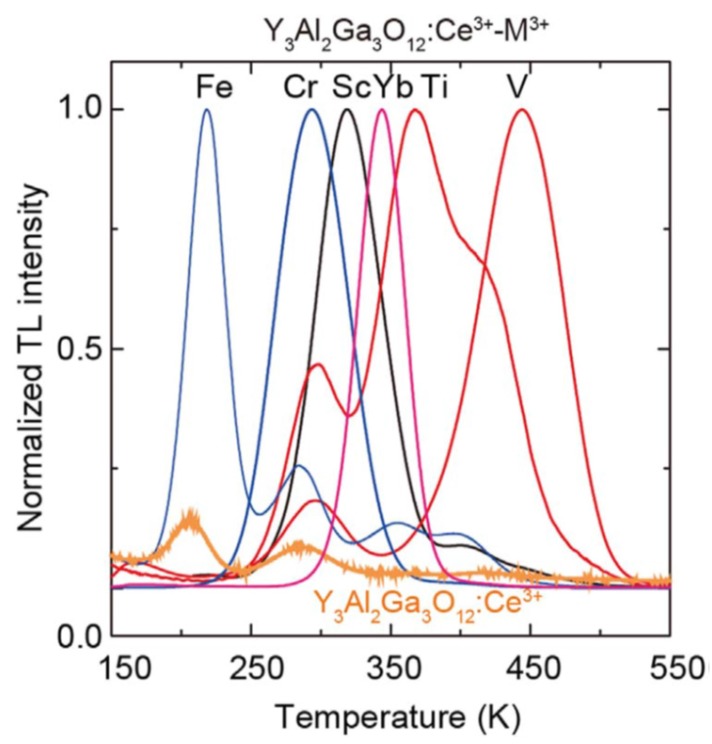
Normalized TL glow curves recorded with a 10 K/min heating rate of Y_3_Al_2_Ga_3_O_12_:Ce^3+^-TM^3+^ (TM = Sc, Ti, V, Cr, Fe), Y_3_Al_2_Ga_3_O_12_:Ce^3+^-Yb_3+_, and Y_3_Al_2_Ga_3_O_12_:Ce^3+^ after UV charging at 100 K. Reproduced from [77].

Persistent luminescence, also called afterglow, is the phenomenon whereby luminescence can last for a certain period of time, from seconds to many hours, after stopping the excitation. The luminescence mechanism is similar to that for thermoluminescence. In fact, persistent luminescence can be considered as TL at room temperature. To design new persistent phosphors, it is important to know the role of the defects (donor or acceptor) and the energy level location. In the most well-known afterglow phosphor, SrAl_2_O_4_:Eu^2+^, Dy^3+^ [76], the Eu^2+^ ions are the recombination centers, while Dy^3+^ plays a role in the trapping. Other trivalent lanthanide (Ln) ions are used as co-dopant, which results in other persistent luminescence properties. Similarly, transition metal ions (TM^2+^) with different *d* electrons can be used. Ueda et al. [77] have carried out a systematic study into the role of TM^3+^, TM = Sc, Ti, V, Cr, Fe in Y_3_Al_2_Ga_3_O_12_:Ce^3^,TM^3+^, where Ce^3+^ is a hole trapping center. The TL glow curves for the different co-dopants are shown in Figure 10. It shows that they act as suitable electron traps like co-dopants of trivalent lanthanides ions in YPO_4_:Ce^3+^,Ln^3+^. Again, TL is shown to be a sensitive technique to determine energy levels in the band gap and is therefore widely used in the study of persistent luminescence phosphors, as illustrated by the many publications in this field [28,29,78,79,80,81,82,83,84,85,86,87,88,89,90,91,92,93,94,95,96]. 

### 4.3. Band Gap Engineering

Afterglow is the property of interest in persistent luminescent phosphors. In the development of new phosphors, one will try to improve the luminescent intensity and extend the duration. However, in a scintillator material, this property is responsible for degradation of the scintillation performance.

**Figure 11:**
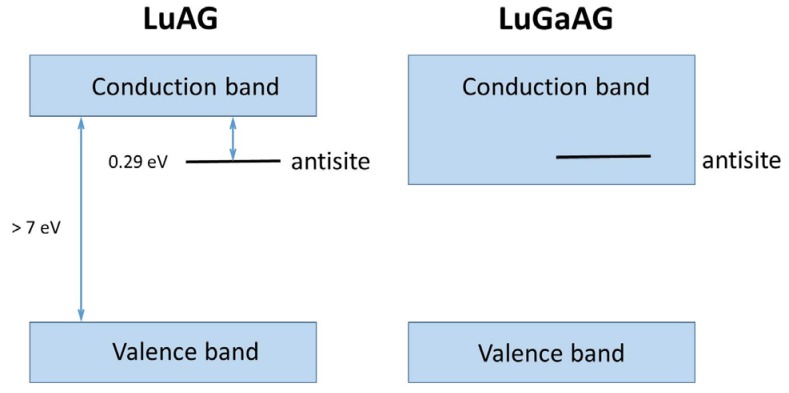
Schematic of the band structure of undoped LuAG (left) with the antisite defect in the band gap 0.29 eV below the conduction band, compared to the band shift due to Ga admix, where the defect level is no longer in the forbidden gap (From [97]).

In LuAG:Ce, for example, TL measurements suggest that the cation antisites induce shallow traps responsible for the afterglow [98]. To suppress the effect of these shallow traps, Fasoli et al. applied a technique called band gap engineering [97]. When LuAG is admixed with Ga^3+^, the band gap gets smaller and the defect level is enveloped by the conduction band (see Figure 11). This clearly improved the scintillator response time. To investigate the conduction band shift as a consequence of the Ga admix, TSL measurements were crucial.

**Figure 12:**
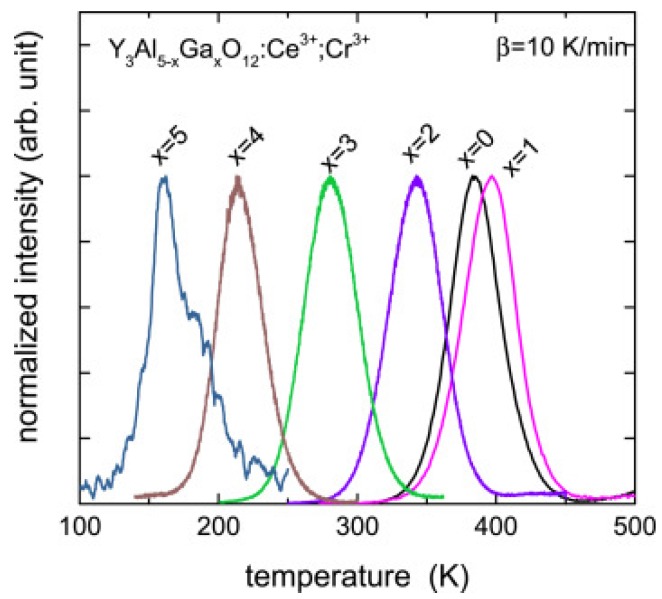
TL glow curves recorded at a heating rate of β = 10 K/min for Y_3_Al_5−*x*_ Ga*_x_*O_12_:Ce^3+^; Cr^3+^ with *x* = 0, 1, 2, 3, 4, and 5, where Ce^3+^ emission is monitored and electrons are released from Cr^2+^. Reproduced from Reference [99] with permission from The Royal Society of Chemistry.

The same technique has been applied in Y_3_Al_5−*x*_ Ga*_x_*O_12_:Ce^3+^;Cr^3+^ samples to control the electron transfer between Ce^3+^ and Cr^3+^ [99]. In Figure 12, the TL glow curves are shown for different values *x* of the Ga content. The observed single TL peak can be attributed to the electron trap of Cr^3+^ (Cr^3+^ + e^−^), which is regarded as the state of Cr^2+^. The peak temperature of the TL glow curve, except for the *x* = 0 sample, lowers with increasing Ga content. From Figure 12, it is seen that that the glow curves do not show the shape of the asymmetric first-order glow peak. Ueda et al. explained this behaviour by assuming that the traps in YAGG:Ce-Cr do not have a single depth, but show a trap depth distribution with a width in energy that depends on the Ga content *x.* The trap depth distribution is assumed to be caused by variations in the environment of the defect responsible for the trap. From simulating the distribution, the authors were able to conclude that with increasing *x*, Ga first starts to occupy the tetrahedral sites, and above *x* = 3, the octahedral sites are also occupied. Katayama et al. [100] reproduced this result in the same material, but it was co-doped with Bi^3+^. Luo et al. [101] studied multicomponent garnet scintillators (Lu*_x_*Gd_3−*x*_)(Ga_*y*_Al_5−*y*_)O_12_:Ce (*x* = 0, 1, 2, 3 and *y* = 0, 1, 2, 3, 4) and identified changes in the band gap by monitoring the shift of the corresponding TL glow peaks. All these studies show that TSL is an indispensable experimental tool in understanding the electron transfer mechanism.

### 4.4. Tunnelling

**Figure 13:**
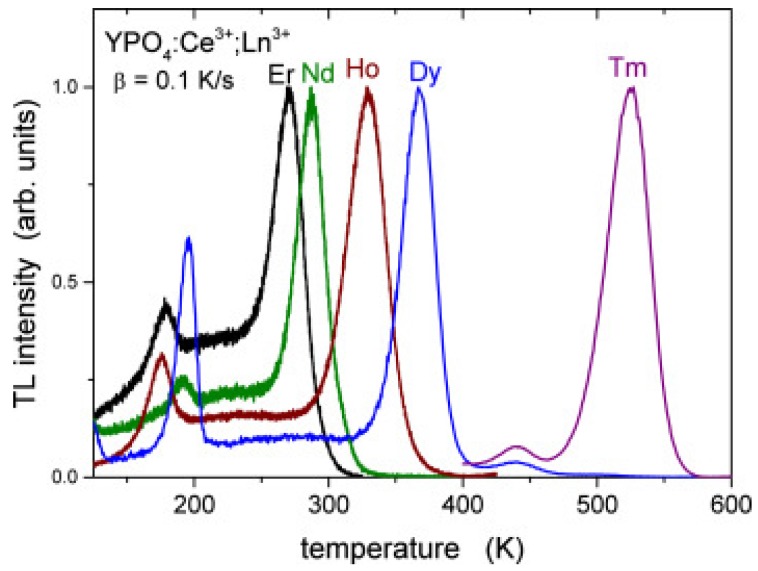
Thermally stimulated glow curves of YPO_4_:Ce^3+^,Ln^3+^ (Ln = Er, Nd, Ho, Dy, and Tm). The plateau before the main peak of the samples with co-dopants Er, Nd, Ho, and Dy is caused by tunnelling recombination (localized transition). The main peaks are caused by recombination via the conduction band (non-localized transition).

In the simple model of thermoluminescence (Figure 1), it is assumed that the recombination pathway of the electron goes via the conduction band. However, an alternative pathway is a localized transition where the electron recombines with a nearby hole center via quantum tunnelling. Tunnelling recombination of trapped electrons with holes has been shown in calcite [102] and natural feldspars [103], but also in synthetic phosphors as Zn_2_SiO_4_:Mn [104] and KCl [105]. The tunnelling process is in principle temperature independent. This is illustrated in Figure 13. For YPO_4_:Ce^3+^,Ln^3+^, the Ce^3+^ ion is the stable hole center and the Ln-copants act as trapping centers. The plateau before the main TL glow peak looks like a background, but is in fact temperature independent luminescence caused by a localized transition. No “background” is observed on the high temperature side of the glow peak because the trap is then fully emptied. Quantum tunnelling may be temperature independent but is critically dependent on the distance between the hole and trapping center. So one might expect that this plateau will rise as more hole and trapping centers are available, decreasing the average distance between the recombination center (Ce^4+^) and trapping center (Ln^2+^); in other words, as the concentration of the dopants increases. This has been investigated by Dobrowolska et al. [106]. They developed a model that successfully explained the shape and behaviour of the TL glow curves as a function of the concentration. 

**Figure 14:**
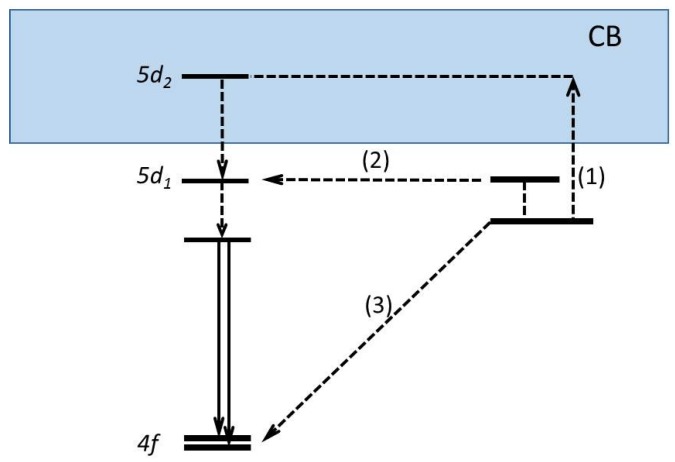
Schematic energy level diagram for the possible recombination pathways of trapped electrons in GAGG:Ce crystal: (1) thermally stimulated non-localized transition (classical TSL), (2) thermally assisted tunnelling, and (3) ground state tunnelling recombination. CB = Conduction Band (Adopted from [107]).

Another recombination pathway is via an excited state of the trapping centre. This is illustrated in Figure 14 for Ce-doped Gd_3_Al_2_Ga_3_O_12_ [107]. The population of the excited state is by thermal activation, causing the thermally assisted tunnelling to become temperature dependent. Thermally assisted tunnelling is also described by Vedda et al. [108], who studied the glow curves from Lu_x_Y_2−x_SiO_5_:Ce. They show that oxygen vacancies act as electron traps in the material. The presence of several glow peaks with a unique trap depth (0.99 eV ± 0.07 eV) for the 78, 135, 181, and 236 °C peaks is explained by suggesting that electrons stored in oxygen vacancies recombine through a thermally assisted tunnelling mechanism with holes localized at Ce^3+^ centers residing on Lu sites at different crystallographic distances from the traps. Nowadays, localised recombinations are recognized in many materials and experimentally and theoretically investigated [109,110,111,112]. 

### 4.5. Thermal Quenching

In many phosphors, the luminescent properties depend on the temperature. At a higher temperature, the luminescence can be quenched. For LED phosphors, for example, the thermal quenching temperature of the luminescence is a crucial parameter. Study of the thermal quenching will usually be performed by measuring the photoluminescence as a function of the temperature. But study of the thermally stimulated luminescence glow curve can give additional information. Glow curves measured at different heating rates will produce a glow peak that shifts to a higher temperature (see Figure 3), but the total area will remain constant. However, if there is thermal quenching, the integrated intensity will decrease. Because the low temperature side of the peak will be less affected than the high temperature side, the shape of the peak will be distorted [113]. From variable heating rate measurements, the quenching function can be derived [114]. In some cases, the temperature integrated luminescence intensity increases with an increasing heating rate [106,115]. This so-called anomalous heating rate dependence can be explained by a model that takes into account both localized and non-localized transitions [116]. Through thermoluminescence excitation spectroscopy, insight into the thermal quenching mechanism can be derived [117].

### 4.6. Photosynthesis

Thermally stimulated luminescence can also be observed from biological material. Arnhold and Sherwood [118] were the first who described TL in chloroplasts. The original discovery of TL in chloroplasts later proved to be a phenomenon common to all photosynthetic organisms. Following the initial observations, considerable effort has been devoted to the identification and characterization of photosynthetic TL components. Nowadays, TL is applied as a research tool to study photosynthetic electron transport under various physiological conditions [119]. As mentioned earlier, the technique requires special instrumental features [46] and the theory described in Section 2, stemming from solid-state physics, needs another theoretical framework [44]. Characteristic for photosynthetic systems is recombination that takes place through various routes among which the radiative pathway generally represents a relatively minor share. The radiative route is the one with the largest activation energy and is thus almost absent at a low temperature. Since its discovery, the origin of many TL components have been identified, characterised, and assigned to the redox components of the Photosystem II, which makes TL a versatile tool in photosynthesis research [45,120,121].

## 5. Conclusions

Thermally stimulated luminescence is a well-known phenomenon that has been studied for a long time. The measurement of the luminescence is purely optical, showing a high sensitivity, and is applicable to both crystalline and non-crystalline material. The first commercially available TL readers were developed for dosimetry and dating purposes. Nowadays, available readers are also suited for research on luminescent materials and are equipped with different detectors, optical filters, and excitation sources. 

The basic explanation of the phenomenon was given by Randall and Wilkins more than 70 years ago. Their model is an oversimplification of a rather complicated situation. Since then, many extensions of the simple model have been given, and nowadays, the theory is being developed to a highly sophisticated level. Each extension of the theory implies an extension of the number of parameters. It has become clear that it is difficult, if not impossible, to extract all parameters. However, one can determine the value of the trap depth with a good accuracy without knowing the other parameters. Applied in combination with other spectroscopic methods like optical absorption emission and excitation spectroscopy, it is highly effective in sensing changes in energy level positions which are crucial for so many luminescent properties. 

In the field of luminescence spectroscopy, a wealth of data have been published during the last 70 years. Extensive investigation of all those data have revealed all sorts of trends in energy levels which can be described in semi-empirical models. TL has proven to be the experimental technique for testing those models. 

In this review, many examples have been given that show that TL is a powerful tool to study material characteristics and a great help in understanding the underlying mechanism of luminescence, as well as processes affecting light production.
